# Pressurized Intraperitoneal Aerosol Chemotherapy (PIPAC): Occupational Health and Safety Aspects

**DOI:** 10.1245/s10434-013-3039-x

**Published:** 2013-06-14

**Authors:** Wiebke Solaß, Urs Giger-Pabst, Jürgen Zieren, Marc A. Reymond

**Affiliations:** 1 Department of Pathology, Ruhr University Bochum, Bochum, Germany; 2 Department of Surgery, Ruhr University Bochum, Bochum, Germany

## Abstract

**Background:**

Pressurized intraperitoneal aerosol chemotherapy (PIPAC) is a novel approach for treating peritoneal carcinomatosis. First encouraging results have been obtained in human patients. However, delivering chemotherapy as an aerosol might result in an increased risk of exposure to health care workers, as compared with other administration routes.

**Methods:**

PIPAC was applied in two human patients using chemotherapeutic drugs (doxorubicin and cisplatin), and air contamination levels were measured under real clinical conditions. Air was collected on a cellulose nitrate filter with a flow of 22.5 m^3^/h. To exclude any risk for health care workers, both procedures were remote controlled. Toxicological research of cisplatin was performed according to NIOSH 7300 protocol. Sampling and analysis were performed by an independent certification organization.

**Results:**

The following safety measures were implemented: closed abdomen, laminar airflow, controlled aerosol waste, and protection curtain. No cisplatin was detected in the air (detection limit < 0.000009 mg/m^3^) at the working positions of the surgeon and the anesthesiologist under real PIPAC conditions.

**Conclusions:**

For the drugs tested, PIPAC is in compliance with European Community working safety law and regulations. Workplace contamination remains below the tolerance margin. The safety measures and conditions as defined above are sufficient. Further protecting devices, such as particulate (air purifying) masks, are not necessary. PIPAC can be used safely in the clinical setting if the conditions specified above are met. However, a toxicological workplace analysis must be performed to confirm that the procedure as implemented complies with local regulations.

Local drug administration has been used as a therapeutic modality for many years and for a broad spectrum of indications. In particular, intraperitoneal chemotherapy (IPC) is increasingly used in clinical practice. The goal of IPC is to increase drug exposure of cancer cells within the peritoneal cavity while minimizing systemic toxicity.^1^ Intraperitoneally administered drugs are expected to penetrate directly into the peritoneal nodules, resulting in a high locoregional bioavailability.^2^ For example, during hyperthermic intraperitoneal chemotherapy (HIPEC), a high dose of chemotherapeutic solution is administered directly into the abdominal cavity, in most cases into the open abdomen, at a temperature of approximately 42.5–43 °C. For multiple indications, HIPEC has been shown to be associated with prolonged survival compared to systemic chemotherapy alone.^3^

However, IPC has two major limitations. First, there is a limited depth of drug penetration into the tissue. The limited tissue penetration leads to a rapid drop in drug concentration below the level needed to destroy tumor cells.^4^ Second, much of the residual tumor burden is untreated or undertreated because peritoneal exposure to chemotherapy is poor.

Pressurized intraperitoneal aerosol chemotherapy (PIPAC) is a novel approach that overcomes several limitations of the more conventional IPC method. PIPAC is a particular application of the general principle of therapeutic capnoperitoneum and of aerosolized chemotherapy.^5^,^6^ Instead of distributing the chemotherapeutic substance in the form of a liquid solution into the abdomen, the drug is nebulized with carbon dioxide to create an aerosol. Aerosols consist of two phases: a liquid phase (droplets) and a gaseous phase. According to physical laws, if the size of droplets is small, aerosols behave like a gas. Because a gas distributes homogeneously within a closed space, the drug concentration is expected to be equal within the whole abdominal cavity.

As a second difference between PIPAC versus IPC, the aerosol is applied within the pressurized abdominal cavity so that a pressure gradient is artificially generated between the intraperitoneal and the extraperitoneal space. As a direct consequence, diffusion of liquids and substances through the peritoneum is enhanced. Moreover, the applied intraperitoneal pressure compensates for the interstitial fluid pressure, which impairs drug uptake into solid tumors and contributes to chemotherapy resistance.^7^

Theoretically, both the more even distribution of chemotherapeutic agents within the abdominal cavity and the improved tissue penetration of drugs provide new therapeutic opportunities to increase the efficacy of intraperitoneally applied chemotherapy. This hypothesis has been confirmed in a rodent model: when intra-abdominal pressure was raised, increased intratumoral drug concentration and enhanced tumor cell death with doxorubicin and cisplatin were observed.^8^,^9^ We have made similar observations in the large animal model as well as in human specimens ex vivo.^5^,^10^ Treating peritoneal diseases with aerosolized drugs has a number of advantages. First, aerosolized chemotherapy provides a direct, minimally invasive means for targeted delivery to different regions of the peritoneum. Second, this route of administration delivers a high dose to the target site. Third, aerosolized IPC causes fewer adverse effects than intravenous administration. Thus, PIPAC opens new avenues in the therapy of peritoneal carcinomatosis, an unmet medical need.^11^

However, delivering chemotherapy as an aerosol might cause an increased risk of exposure to health care workers compared to other administration routes. This is due to the difficulty of controlling the spread of aerosols during PIPAC, which in turn contributes to the risk of leakage and unwanted exposition.

To prevent any harm to health care workers, we have identified and evaluated potential hazards concerning occupational exposures during PIPAC performance. In a second set of experiments, we have simulated PIPAC in the laboratory and in the operating room (OR). In addition, we have applied PIPAC in the human patient using chemotherapeutic drugs and measured contamination levels under real clinical conditions.

## Methods

### Ethical, Legal, and Regulatory Background

The study protocol was submitted to the institutional review board (IRB; Common Ethics Committee of the Westfalian Wilhelms-University Münster and of the Westfalian Medical Chamber). The IRB recommended performing the first PIPAC therapy with volunteers, which were extensively informed and trained in the PIPAC procedure.

### Methodology

The following steps were defined: identification of hazardous substances and dose; identification of possible exposure ways; simulation of the PIPAC procedure with nontoxic aerosols and smoke; redaction of standard operating procedures (SOP); second simulation according to the SOP; informing and training the health care workers; and performance of the first two PIPAC procedures with chemotherapeutic substances and workplace measurements under real conditions.

### Nebulizer

The nebulizer (Reger Medizintechnik, Rottweil, Germany) has been described elsewhere .^5^ In brief, it consists of several components, including an injector, a tube, and a nozzle. The nozzle has a diameter of 0.2 mm. A pressure of up to 20 bar is delivered upstream of the nozzle, using an industry-standard contrast medium injector (Injektron 82 M, MedTron, Saarbrücken, Germany, including a remote control device, MT1130/1).

### Operating Room Characteristics

The PIPAC procedure was performed within an OR equipped with laminar airflow. Volume of the OR was approximately 168 m^3^. Air flow was 1.8 × 10^6^ L/h. Room temperature was 22.3–22.6 °C. Relative humidity was 36–37 %. Atmospheric pressure was 994 hPa. Vacuum was generated with a pressure of −0.85 bar (Dräger, Lübeck, Germany).

### Assumptions

The assumptions for the determination of exposure are listed in Table 1.

**Table 1:** Assumptions for determination of exposure

Parameter	Value
Room temperature	22 °C
Inhalation rate	1.5 m^3^/h
Body weight	70 kg
Body surface	1.7 m^2^
Duration of therapy	30–60 min per application; 1 therapy per day
Total amount of applied CO_2_	3 to max. 6 L
Duration of exposure (presence of OR team)	No routine presence; if intervention required, max. 20 min per procedure
Technical details of application	Pressure in injector: 0.8 bar
	Pressure at the nozzle: 0 bar
	Duration of nebulizing: ~5 min
	Total applied volume: 150 ml per chemotherapeutic cycle
	Diameter of nozzle: 0.2 mm

### Chemotherapy

We have focused on the application of two chemotherapeutic agents: cisplatin and doxorubicin. Chemotherapy was applied as follows: nebulization over 3–6 min of 7.5 mg cisplatin/m^2^ body surface followed immediately by the nebulization of 1.5 mg doxorubicin/m^2^ body surface into the abdominal cavity filled with CO_2_ at a pressure of 16–20 mbar (12–15 mm Hg) at a temperature of 37 °C followed by 30 min steady-state before exsufflation.

### Experimental Protocol

Two PIPAC procedures were performed in two consecutive patients within the same OR. Between the procedures, the room was cleaned according to the hospital’s standard hygiene and surface cleaning protocols.

Each procedure was structured into four consecutive phases, as follows:

- Phase 1: CO_2_ insufflation is provided over an industry-standard trocar (Kii Access System, Applied Medical, Darmstadt, Germany), with a target pressure of 16 mbar (12 mm Hg). The access system was secured with an intra-abdominal balloon and an extra-abdominal obturator, ensuring tightness of the abdomen and steadiness of the pressure. Two 5-mm working trocars are inserted.
- Phase 2: A nebulizer (MIP, Reger Medizintechnik, Rottweil, Germany) was introduced through the access trocar and aerosol formation of the chemotherapy solution into the abdominal cavity using the injector over.
- Phase 3: The system was kept in steady state for 30 min at a constant pressure and temperature. The abdomen was hermetically sealed; the total gas flow was minimal.
- Phase 4: At the end of the procedure, the gas from the abdomen was released directly into the hospital’s waste-air system over one of the trocars and an aerosol/smoke filter (pores 0.027 μm, model 03110-10, mtp, Neuhausenob Eck, Germany).

### Toxicology Analysis

The probe sampling system used was a Gravikon VC25 device combined with a dust detector (Ströhlein, Kaarst, Germany). Air was collected on a cellulose nitrate filter with a diameter of 50 mm, with a flow of 22.5 m^3^/h. Toxicological research analysis of cisplatin levels was performed according to a standard protocol (NIOSH 7300). The detection limit was 0.3 μg/sample. Sampling and analysis were performed by engineers of the Division for Hazardous Substances at the Laboratory for Environmental and Product Analysis of DEKRA Industrial GmbH in Stuttgart (Germany), an independent certification organization.

## Results

### Identification of Hazardous Substances and Dose

The toxicological characteristics of cisplatin and doxorubicin are summarized in Table 2. In short, cisplatin is highly poisonous. It can provoke anaphylactic reactions and it irritates the eyes and skin; it has no transdermal absorption. Furthermore, it irritates airways and has a cumulative toxic effect on kidney, bone marrow, and the inner ear. It is probably carcinogenic to humans. Doxorubicin is hazardous to human health by provoking mucosal inflammation, leucopenia, and dilative cardiomyopathy. Additionally, it induces DNA mutation and is carcinogenic to humans. The total dose applied during PIPAC is approximately 10 % of a usual systemic chemotherapy dose. There is no legal exposure limit for either of these two substances in Germany. However, in the Netherlands, the maximally allowed air concentration for cisplatin is <0.00005 mg/m^3^.

**Table 2:** Safety data for cisplatin and doxorubicin

Parameter	Cisplatin	Doxorubicin
CAS-/EG no.	15663-27-1/239-733-8	23214-92-8/245-495-6
		25316-40-9/246-818-3 (hydrochloride)
Formula		
Molecular weight	300.06 g/mol	543.52 g/mol
Melting point	270 °C; dark yellow powder at room temperature	205 °C (degradation); crystalline red powder at room temperature
Boiling point	Not applicable	Not applicable
Steam pressure	Not applicable	Not applicable
Water solubility	2.530 g/L (25 °C)	0.0928 g/L (25 °C)
LD50 oral	20 mg/kg (rat)	570 mg/kg (mouse)
	32 mg/kg (mouse)	–
NOAEL	No data	No data
Important toxicological details	Acute toxicity: very toxic Skin and eye irritation No evidence for transdermal absorption Cumulative damage of kidney, bone marrow, and inner ear No evidence of carcinogenicity in human Evidence for carcinogenicity and teratogenicity in mouse and rat Level of carcinogenicity: 2A Anaphylactic reactions reported Sensibilization of skin and airways	Acute toxicity: harmful Dilatative cardiomyopathy Inflammation of mucosa Leucopenia Evidence for carcinogenicity in animals Evidence for mutagenicity in animals Level of carcinogenicity 2A
Total amount applied	15 mg in 150 ml NaCl 0.9 %	3 mg in 150 ml NaCl 0.9 % solution
Concentration of applied solution	0.1 mg/ml = 0.1 g/L = 0.01 %	0.02 mg/ml = 0.02 g/L = 0.002 %
Workplace exposure limits	Germany: not available	Germany no upper legal limit
	Netherlands: 0.00005 mg/m^3^	–

### Identification of Possible Mean of Exposure

The preparation of the chemotherapeutic agents in the hospital pharmacy and their transport in adequate containers to the OR is scheduled according to the German recommendations.^12^ Both agents are provided in a closed delivery system (special injection syringes filled with NaCl 0.9 % solution). Identified exposure ways are ocular, dermal, and inhalative exposition. Other possibilities were reasonably excluded.

### First PIPAC Simulation with NaCl 0.9 % Solution

Before performing the first clinical PIPAC application, the procedure was simulated in the OR using a laparoscopy training phantom and NaCl 0.9 % aerosol. Working steps were written down, and risk analysis was performed within an interdisciplinary team including physicians (surgeons and anesthesiologists), scrub nurses, hospital technicians, nebulizer engineers, and occupational health experts. Possible mechanical failures related to the injector, the infusion tube, the nebulizer itself, the laminar flow system, and the tightness of the abdomen were identified. In case of any failure of the procedure, appropriate security measures were taken and defined. A standard operating protocol was thus established that served as a basis for the second simulation.

### Second PIPAC Simulation with Smoke and Artificial Leak

**Fig. 1:**
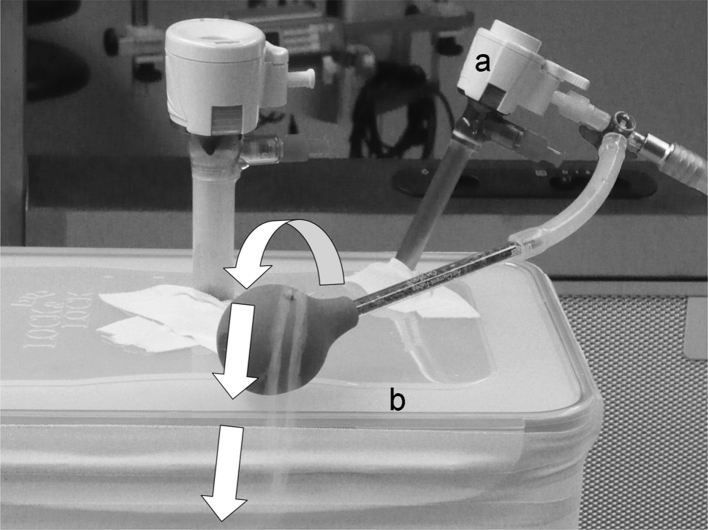
PIPAC simulation with smoke and artificial leakage. Sealing access trocars (**a**) were introduced into a sealed plastic box (**b**) with the same volume dimensions as the human abdominal cavity. The *box* was pressurized with CO_2_ and steam. Via an artificial leakage (open access trocar), the steam (*white bold arrows*) was observed to be directed to the floor and not randomly distributed within the OR. This is caused by the laminar air flowing downward from the ceiling to the floor

The second simulation was performed in the OR under strict implementation of the SOP simulating the abdomen with a sealed plastic container of similar dimensions. An aerosol of CO_2_ and smoke with the same pressure as during laparoscopy (using identical, industry-standard technical instruments, such as access trocars, video camera, grasping forceps) was applied. We were able to perform the complete procedure without any incident; in particular, the system remained tight. Then a maximal leakage (an access trocar was fully opened) was simulated. The smoke escaping from the leak was flowing downward (Fig. 1) to the floor and into the lateral outflow windows of the hospital air-waste system.

### Information and Training of the Team of Volunteers

On the basis of the successful simulations, it was decided to schedule two patients for the first PIPAC procedures. Informational meetings allowing open, interactive discussion were organized because the planned procedures raised emotional concerns, in particular among scrub nurses and cleaning workers. On the basis of these discussions, we decided to restrict the first procedure to volunteers within the framework of a special shift that excluded other simultaneous surgical procedures. Before the first procedure, the team of volunteers received interdisciplinary training according to the SOP.

### Performance of First PIPAC Procedures with Chemotherapy

**Fig. 2:**
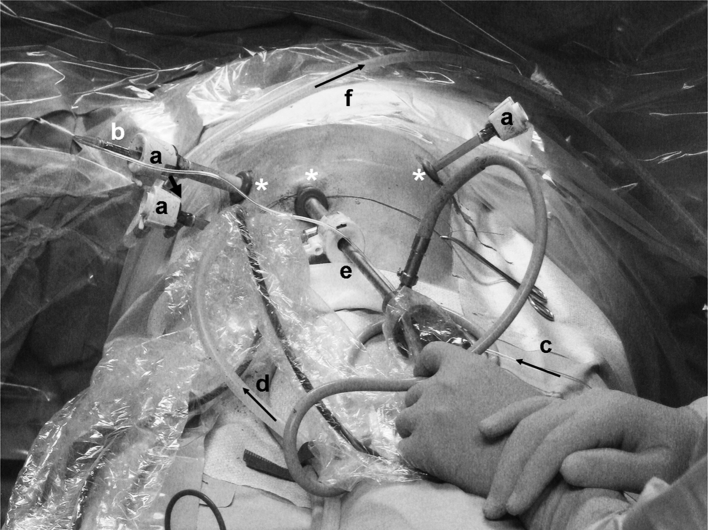
First PIPAC under real conditions. Access trocars (**a**) with the nebulizer (**b**) in situ. The chemotherapeutic agents were transported from the injector to the nebulizer via a high-pressure infusion line (**c**). CO_2_ was injected into the abdominal cavity via a standard gas line (**d**) and the trocar (**e**) (camera trocar). At the end of the procedure, the chemotherapeutic capnoperitoneum was desufflated via line (**f**) over an aerosol filter into the air-waste system of the hospital. *Dark arrows* indicate the flow direction of the gas and chemotherapeutics. *Asterisk* Trocar sealing rings

The first PIPAC procedure was performed on November 5, 2011, under the supervision of a safety officer and included workplace air measurements. The SOP were strictly implemented; in particular, nobody remained within the OR during the PIPAC procedure, which was remote controlled. The nebulizer functioned as expected, and the system remained airtight (Fig. 2). At the end of the procedure, the chemotherapy aerosol was exhausted into the air-waste system of the hospital and released into the environment.

### Air Contamination of the Operating Room with Cisplatin

**FIG. 3:**
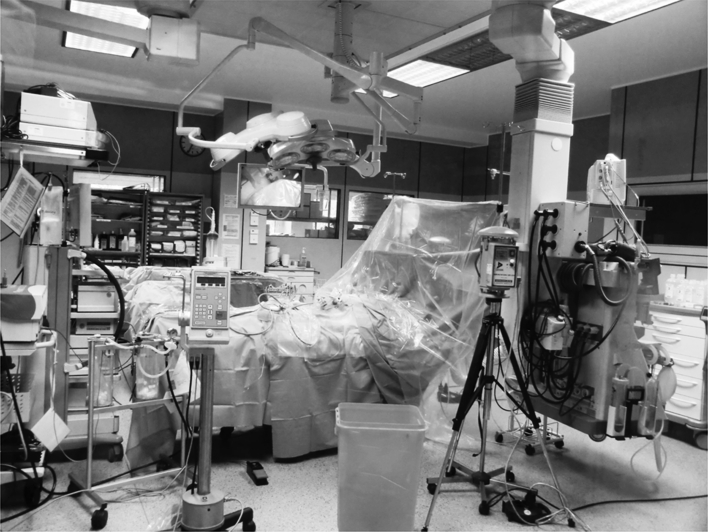
OR setup for first PIPAC and safety measurement. The OR is equipped with laminar airflow. The abdomen is tight. The procedure is remote controlled. Environmental air sampling was undertaken at the surgeon’s (**a**) and anesthesiologist’s (**b**) working positions. The pressure injector (**c**), sealing trocars, and nebulizer in situ (**d**) as well as the desufflation line (**e**) are shown. To minimize any possible chemotherapeutic exposure of the anesthesiology crew, a vertical transparent curtain dividing the laminar airflow was hung between the patient’s head and the site of chemotherapy application

Air was sampled during two consecutive PIPAC procedures (Fig. 3). Results are summarized in Table 3. Air analysis revealed no traces of cisplatin, either at the position of the surgeon or the anesthesiologist.

**Table 3:** Measurement of cisplatin concentration in the operating room

Characteristic	Measurement location^a^	
	1	2
Probe no.	0511-01	0511-02
Date	05.11.2011	05.11.2011
Start (hh:mm)	12:15	12:15
End (hh:mm)	16:38	16:38
Duration (h)	2.4	2.4
Air pressure (hPa)	994	994
Temperature (°C)	22.5	22.5
Volume stream (m^3^/h)	22.5	22.5
Partial gas volume (m^3^)	54.2	54.0
Platin in inhalable dust (mg/Pr.)	0.3	0.3
LoD (mg/Pr.)	0.3	0.3
Relative LoD (mg/m^3^)	0.000006	0.000006
Analysis (mg/Pr.)	<0.3	<0.3
Concentration (mg/m^3^)	<0.000006	<0.000006
Platin calculated as cisplatin concentration (mg/m^3^)	<0.000009	<0.000009

To allow precise analysis of the limit of determination (LoD), probe sampling was performed during 2 operations, but only when chemotherapeutic drugs were administered. In the meantime, sampling was paused

*LoD* limit of determination

^a^Measurement locations: *1* anesthesiologist’s position (patient’s head), *2* surgeon’s position (patient’s abdomen)

### Conclusions

Chemotherapy is an essential component of modern multimodal cancer therapy. However, many drugs used to fight cancer are mutagenic, teratogenic, and carcinogenic in experimental systems.

Development of PIPAC has raised concerns about the risk of occupational inhalation linked to the application of toxic aerosols. To assess this problem, it was not possible to rely on existing safety standards because PIPAC had never yet been performed. However, we could compare—to some extent—intraperitoneal aerosol chemotherapy to aerosolized chemotherapy in lung cancer. In this latter setting, aerosolized chemotherapy has been delivered in a well-ventilated room with an air-filtering system.^13^ Alternatively, a mobile-filter air-cleaning system combined with a collecting tent was also effective in preventing propagation of aerosol during inhalation of nebulized liposomal cisplatin.^14^ Chemotherapy levels in the air were below workplace exposure limits. Other recent phase 1 studies have demonstrated the feasibility and safety of aerosol delivery of doxorubicin and gemcitabine in lung cancer patients.^14^,^15^

In occupational settings, environmental monitoring of exposure to toxic aerosols seems to be superior to biological monitoring. It offers the possibility of simultaneous determination of components of mixtures, is simple to interpret, and evaluates short-term exposure to environmental irritants.^16^ Thus, estimation of exposure under real conditions was an important step to provide a safe working environment during PIPAC. The total chemotherapy dose was 1:10 of a systemic dose delivered intravenously. The aerosol was applied within the closed abdomen, and no leakage occurred. No cisplatin contamination in the air was detected.

Because PIPAC is applied within a closed system, the risk of skin contamination with chemotherapy is also minimal (e.g., after a manipulation error with the contrast medium injector, or use of inadequate, low-pressure infusion tubing). This risk can be reduced by providing one-block systems (nebulizer and infusion tubing), and by training and drilling in order to minimize human errors. In the case of leakage, skin contamination with chemotherapy solutions would be adequately met by wearing special chemotherapy gloves and protective glasses. A special set of gloves used to remove spilled chemotherapy solution is available on the OR. The OR has to be cleaned afterward, as it is routinely the case because of biological risks such as blood contamination. Tissues, tubes, lines, and other devices such as operation drapes and sponges have to be disposed into special sealed, labeled containers.

In summary, this study shows that the risk of occupational exposure to chemotherapy during PIPAC has been reduced to a minimum so that the procedure complies with German occupational safety regulations. This is an important precondition for beginning phase 2 and phase 3 clinical studies in order to define the possibilities and limits of PIPAC in the therapy of peritoneal carcinomatosis. Strict application of the SOP, repeated measurement of exposure levels, and continuous education of physicians and nurses will be necessary with the increasing use of this new therapeutic strategy in order to avoid any harm.

After the implementation of all equipment, organizational aspects, and procedures as described above, any other team starting PIPAC should perform a toxicological workplace analysis. This analysis must to be scheduled before the routine application of PIPAC to ensure that it can be performed in accordance with local regulations.

## What This Study Adds

It is possible to apply a therapeutic pressurized chemotherapy aerosol into the abdominal cavity of peritoneal carcinomatosis patients without occupational health hazards.

## References

[1] Markman M (2003). Intraperitoneal antineoplastic drug delivery: rationale and results. Lancet Oncol..

[2] Alberts DS, Young L, Mason N (1985). In vitro evaluation of anticancer drugs against ovarian cancer at concentrations achievable by intraperitoneal administration. Semin Oncol..

[3] Verwaal VJ, Bruin S, Boot H (2008). 8-year follow-up of randomized trial: cytoreduction and hyperthermic intraperitoneal chemotherapy versus systemic chemotherapy in patients with peritoneal carcinomatosis of colorectal cancer. Ann Surg Oncol..

[4] Trédan O, Galmarini CM, Patel K (2007). Drug resistance and the solid tumor microenvironment. J Natl Cancer Inst..

[5] Solaß W, Hetzel A, Nadiradze G (2012). Description of a novel approach for intraperitoneal drug delivery and the related device. Surg Endosc..

[6] Laube BL (2005). The expanding role of aerosols in systemic drug delivery, genetherapy, and vaccination. Respir Care..

[7] Minchinton AI, Tannock IF (2006). Drug penetration in solid tumors. Nat Rev Cancer..

[8] Jacquet P, Stuart OA, Chang D (1996). Effects of intra-abdominal pressure on pharmacokinetics and tissue distribution of doxorubicin after intraperitoneal administration. Anticancer Drugs..

[9] Esquis P, Consolo D, Magnin G (2006). High intra-abdominal pressure enhances the penetration and antitumor effect of intraperitoneal cisplatin on experimental peritoneal carcinomatosis. Ann Surg..

[10] Solass W, Herbette A, Schwarz T (2012). Therapeutic approach of human peritoneal carcinomatosis with Dbait in combination with capnoperitoneum: proof of concept. Surg Endosc..

[11] Canis M, Matsuzaki S, Bourdel N (2007). Peritoneum and laparoscopic environment. Bull Cancer..

[12] Zytostatika im Gesundheitsdienst. Informatiuon zur sicheren Handhabung von Zytostatika. GUV-I 8533. Berlin: Deutsche Gesetzliche Unfallversicherung; 2008.

[13] Wittgen BP, Kunst PW, Perkins WR (2006). Assessing a system to capture stray aerosol during inhalation of nebulized liposomal cisplatin. J Aerosol Med..

[14] Otterson GA, Villalona-Calero MA, Sharma S (2007). Phase I study of inhaled doxorubicin for patients with metastatic tumors to the lungs. Clin Cancer Res..

[15] Lemarie E, Vecellio L, Hureaux J (2011). Aerosolized gemcitabine in patients with carcinoma of the lung: feasibility and safety study. J Aerosol Med Pulm Drug Deliv..

[16] Jakubowski M (2012). Biological monitoring versus air monitoring strategies in assessing environmental-occupational exposure. J Environ Monit..

